# Pravastatin in preeclampsia: A meta-analysis and systematic review

**DOI:** 10.3389/fmed.2022.1076372

**Published:** 2023-01-13

**Authors:** Balázs Mészáros, Dániel Sándor Veres, Luca Nagyistók, Anikó Somogyi, Klára Rosta, Zoltán Herold, Zoltán Kukor, Sándor Valent

**Affiliations:** ^1^Department of Obstetrics and Gynecology, Semmelweis University, Budapest, Hungary; ^2^Department of Biophysics and Radiation Biology, Semmelweis University, Budapest, Hungary; ^3^Dél-Pest Centrum Hospital National Hematology and Infectious Diseases Institute, Budapest, Hungary; ^4^Department of Internal Medicine and Haematology, Semmelweis University, Budapest, Hungary; ^5^Department of Obstetrics and Gynecology, Medical University of Vienna, Vienna, Austria; ^6^Division of Oncology, Department of Internal Medicine and Oncology, Semmelweis University, Budapest, Hungary; ^7^Department of Molecular Biology, Institute of Biochemistry and Molecular Biology, Semmelweis University, Budapest, Hungary

**Keywords:** preeclampsia, statin, pravastatin, meta-analysis, systematic review, prevention, intrauterine growth restriction (IUGR), neonatal intensive care unit (NICU)

## Abstract

**Objective:**

To review of the efficacy and safety of pravastatin use for prophylaxis and treatment of preeclampsia.

**Design:**

Systematic review and meta-analysis of clinical studies evaluating pravastatin for treatment and/or prophylaxis of preeclampsia.

**Data collection:**

Two independent reviewers systematically searched data from PubMed, Scopus, Web of Science, Cochrane, Embase, and clinicaltrials.gov databases, for studies evaluating pravastatin for prevention of pre-eclampsia.

**Results:**

Fourteen studies were identified, including 1,570 pregnant women who received either pravastatin or placebo, published between 2003 and 2022. From these studies, 5 studies were identified for inclusion in the meta-analysis to evaluate the role of pravastatin use prior to 20 weeks of gestation, to prevent pre-eclampsia, Pravastatin treatment reduced the incidence of preeclampsia by 61% and premature birth by 45%. Among the newborns, there was a 45% reduction in intrauterine growth retardation (IUGR) in the treated group, as well as a 77% reduction in those receiving neonatal intensive care unit (NICU) admissions.

**Conclusion:**

Prophylactic treatment with pravastatin appears to reduce risk of developing pre-eclampsia as well as potentially lowering risk of IUGR, preterm birth, and NICU admission in neonates.

## 1. Introduction

According to the International Society for the Study of Hypertension in Pregnancy (ISSHP), the definition of pre-eclampsia is new-onset hypertension after 20 weeks of gestation, accompanied by proteinuria and/or maternal acute kidney injury (AKI), liver dysfunction, neurological dysfunction, thrombocytopenia or hemolysis, or fetal growth restriction (1). It is a pregnancy-specific disorder that is one of the leading causes of maternal and neonatal morbidity and mortality and has a prevalence of 2–8% worldwide (2, 3).

While the exact pathomechanism of the disease is incompletely understood, most researchers consider it a multifactorial disease that has genetic and an environmental contributory factor (4–6). It is likely underpinned by abnormal placentation which subsequently leads to the release of antiangiogenic markers such as soluble fms-like tyrosine kinase-1 (sFlt-1) and soluble endoglin (sEng). The increased level of sFlt-1 and sEng contribute to endothelial dysfunction, and vasoconstriction, affecting maternal and fetal organs (6–8). According to the latest views, preeclampsia is not a single disease but more precisely a group of conditions that might have slightly different characteristics and pathomechanisms (5, 7).

Currently, the only definitive treatment for preeclampsia is delivery of the baby (2). However, there are a number of treatments which aim to prevent the manifestation of the disease: for instance, low-dose aspirin is widely used as a prophylaxis in high-risk-population (8, 9). However, lately, there has been a growing interest in the prevention and treatment of preeclampsia with statins, especially with pravastatin (5). In our systematic review and meta-analysis, we aimed to evaluate the role of pravastatin in the prevention and treatment of preeclampsia.

The rate-limiting step of cholesterol synthesis is the reduction of HMG-CoA to mevalonate, which reaction is catalyzed by HMG CoA reductase, statins are competitive inhibitors of this enzyme, in this way, they effectively lower the blood level of cholesterol (10, 11). Many studies indicate that statins, especially pravastatin increase the level of PlGF (placental growth factor), which lowers the level of sFlt-1 thus reversing the effects of anti-angiogenic factors which lead to preeclampsia (5, 12). Other studies suggest that pravastatin enhances microsomal arginine uptake thus inducing NO synthesis which has a positive effect on microcirculation (13, 14).

There are both lipophilic and hydrophilic statins. Since pravastatin is a hydrophilic statin, it has favorable pharmacokinetics, and it is less likely to be teratogenic than lipophilic ones. Pravastatin being less teratogenic than lipophilic statins were reported in studies, where females - unaware of their pregnancy–took statins during the first gestational weeks (11, 15). Moreover, recent data suggests statins, regardless of their type, do not cause congenital anomalies (16).

The previously mentioned growing interest in statin therapy in preeclampsia is clearly shown in the PubMed database, if we type in the keywords “statin + preeclampsia” we can see that in the first five years-between 2003 and 2007-only 5 articles were published and in the last 5 full years (2017–2021) 89 studies were published.

### 1.1. Choice of pravastatin

Statins are contraindicated in pregnancy (17), however, a recently published meta-analysis (18) suggested statin therapy to be safe as it was not associated with stillbirth or induced and elective abortion rates. Although, a significant increase after statin therapy was described in the number of spontaneous abortions.

**FIGURE 1:**
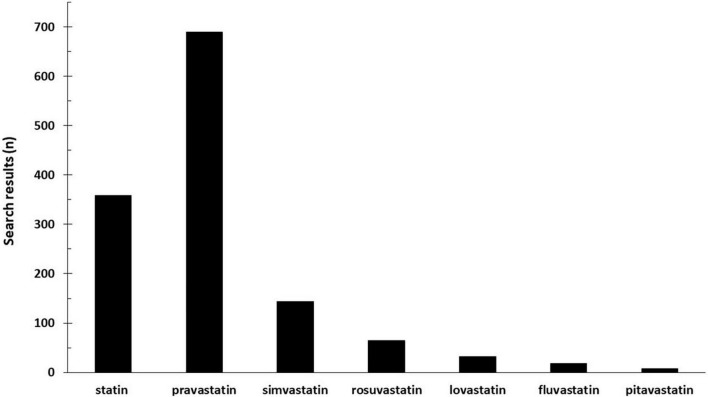
Number of hits for search terms “statin AND preeclampsia” databases: PubMed, Cochrane, Embase, Web of Science, Scopus, and www.clinicaltrials.gov.

The difference in the use of statins in the treatment of preeclampsia is striking. Pravastatin is used almost exclusively in scientific studies (Figure 1). Pravastatin significantly reduced the secretion of both ET-1 and sFlt-1 (key mediators of endothelial dysfunction) in primary human umbilical vein endothelial cells (HUVECs) and uterine microvascular cells (UtMVs) (19). Pravastatin can improve the insufficient NO supply characteristic of preeclampsia (20–22). In a human trophoblast-like cell line (HUVEC), pravastatin increased the expression of endothelial NO-synthase and promoted eNOS activity by phosphorylating the activating eNOS Ser1177 (23).

Simvastatin may be a more potent inhibitor of sFlt-1 secretion from endothelial cells, trophoblast cells, and placenta from women with preterm preeclampsia than either pravastatin or rosuvastatin (24). In a human cell line (Human choriocarcinoma JAR cells), simvastatin reduces oxidative stress, which is the reason it can potentially play a role in the treatment of preeclampsia (25). Simvastatin treatment significantly decreased hypertension, sFlt-1, TNF-α, and oxidative stress marker malondialdehyde levels in a preeclampsia rat model (NG-nitro-L-arginine methyl ester-induced) (26). Pravastatin reduces the reduction of free cytochrome c by glutathione and the oxygen consumption of the mitochondria, while simvastatin increases the reduction of cytochrome c and the mitochondrial oxygen consumption. Simvastatin could enhance the oxidizing capacity of free cytochrome c, thereby increasing oxidative stress and thus facilitating apoptosis (27). Moreover, rat models of preeclampsia were even treated successfully with pravastatin (28). Among the tested statins (pravastatin, simvastatin, rosuvastatin) simvastatin was the most potent inhibitor of sFlt-1 secretion from endothelial cells, trophoblast cells, and preterm preeclamptic placental explants (24). Pravastatin also significantly reduced the secretion of both ET-1, sFlt-1, and other key mediators of endothelial dysfunction in HUVECs. Importantly, pravastatin had no toxic effects, contrary to rosuvastatin and simvastatin (19). The risk is strongly intensified by drug interactions through CYP3A4. Pravastatin is not converted by cytochrome P450, it is excreted after sulfation. Thus, it puts less strain on the liver than other statins. In animal experiments, pravastatin was found to be safe in pregnancy, with no toxic effects. Its beneficial effect has been demonstrated in several preeclampsia models (29).

### 1.2. Objectives

In this study, we performed a systematic review focusing on the efficacy and safety of pravastatin use in preeclampsia. We aimed to investigate the effect of pravastatin treatment in. We investigated whether pravastatin could have a role in the prevention or treatment of preeclampsia in high-risk groups, in terms of maternal and fetal outcomes. Firstly, we evaluated the use of pravastatin prior to 20 weeks of gestation, to prevent pre-eclampsia and performed a meta-analysis of 5 identified studies (Group 1). Secondly, we investigated the use of pravastatin after 20 weeks of gestation to prevent pre-eclampsia (Group 2). Thirdly, we evaluated studies which used pravastatin to treat established pre-eclampsia (Group 3).

## 2. Materials and methods

Two independent reviewers collected data from PubMed, Cochrane, Embase, Web of Science, Scopus, and clinicaltrials.gov databases of studies published in the last 20 years: between 2003 January and 2022 July. We used the keywords “statin” OR “pravastatin” OR “simvastatin” OR “rosuvastatin” OR “lovastatin” OR “pitavastatin” OR “fluvastatin” AND “preeclampsia” as we conducted our study. The search term “*statins” were used in a separate search. Language restrictions were not used.

In this summary, we present the data obtained with the search terms “pravastatin” and “preeclampsia” in detail.

Inclusion criteria: Statin treatment during human pregnancy with an untreated control group for the treatment of preeclampsia or prevention purposes.

Exclusion criteria: Non-human study, summary, case report, *in vitro* study.

After the selection, we divided the studies into the three groups mentioned in the Objectives (1st prevention before 20th week, 2nd prevention after 20th week, and 3rd treatment).

### 2.1. Study selection

Two investigators (LN and BM) determined the eligibility of retrieved studies independently, according to predetermined criteria. Disagreements were resolved by consensus and, if necessary, by the involvement of a third reviewer (ZK).

We only included studies where statins were used in humans, and they evaluated their role in the prevention and/or treatment of preeclampsia.

### 2.2. Data extraction

The following characteristics of the included studies were extracted: authors, year of publication, study design, the objective of the studies, number of study participants (as well as the number of the control group and the number of the placebo group), type and dosage of the used statin, other concurrent medications for the prevention of preeclampsia and gestational weeks of statin exposure. We also extracted the following outcomes if available: maternal and fetal toxicity and adverse effects, birth weight, termination of pregnancy (weeks), neonatal deaths, spontaneous abortions, NICU admissions, and preterm birth.

### 2.3. Statistical methods

A risk ratio (OR) with a 95% confidence interval (CI) was used for the effect size measure. To calculate the risk ratio, the total number of patients and those with the event of interest in each group separately were extracted from the studies where it was available. As we anticipated considerable between-study heterogeneity, a random-effects model was used to pool effect sizes.

As we have studies with small sample sizes and some studies with zero cell counts, we preferred to perform an analysis with the exact Mantel-Haenszel method (without continuity correction) (30, 31) as it is robust for the mentioned situation recommended by J. Sweeting, J. Sutton, and C. Lambert in a 2004 publication (32). We applied a Hartung-Knapp adjustment (31, 33) if it was more conservative compared to without this adjustment.

To estimate the heterogeneity variance measure τ^2^ the Paule-Mandel method (34) was applied with the Q profile method for the confidence interval (35).

Additionally, between-study heterogeneity was described by Higgins and Thompson’s *I*^2^ statistics (36).

Forest plots were used to graphically summarize the results. In the case of zero cell counts, individual OR with 95% CI was calculated by adding 0.5 as continuity correction (it was used only for visualization on Forest plot, for pooling the exact Mantel-Haenszel method was used).

The study number was relatively low, and the heterogeneity was relatively high therefore we did not present the prediction intervals (i.e., the expected range of effects of future studies) on the plots and its meaning is limited. For the already mentioned reasons, the outlier and influence analyses are less powerful.

All statistical analyses were made with R (37) using the meta (38) package.

## 3. Results

### 3.1. Description of studies

#### 3.1.1. Study inclusion for the systematic review

We used our previous work’s database for the analysis (39). The electronic database search between January 2020 and July 2022 provided a total of 313 articles, after the duplicate exclusion, there were 113 articles left. Since most of the articles were dealing with animal models and tissue samples, out of these 83 were not relevant to our meta-analysis. 30 studies were considered for full-text assessment, however, we needed to exclude 14, then another 5 out of the remaining studies: they were either responses for the authors, or they were not primarily focusing on the treatment of preeclampsia with statins and/or did not provide enough data for our research which primary objective was to examine the safety and efficacy of statins in the treatment and prevention of preeclampsia. After the exclusion, 11 articles met the inclusion criteria, we added 3 other articles which were already selected in the author’s earlier database, which had the same object, and it was covering the studies between 2003 and 2016. We examined these articles and extracted data from them. These studies were included in the systematic research because they used pravastatin in the treatment/prevention of preeclampsia–these 14 articles were examined later in the review part of the article.

#### 3.1.2. Study inclusion for the meta-analysis

**FIGURE 2:**
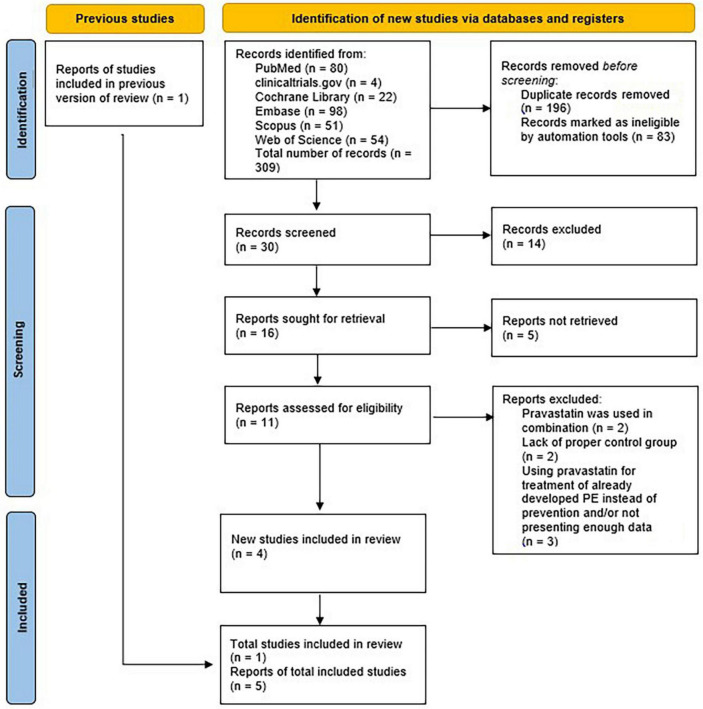
Meta-analysis flow diagram of prevention.

With the already existing data, a meta-analysis was conducted. The aim of it was to evaluate the role of pravastatin in the prevention of preeclampsia before the 20th gestational week. Out of the 11 articles which were the results of the newly done database search, 2 were excluded because pravastatin was used in combination with L-arginine, another 2 because of being case reports, in this way not providing proper control groups and another 3 of them were left out because they were using pravastatin in the treatment of preeclampsia in later gestational weeks and/or were not providing enough data. This way four records were left and 1 was selected from the previous database, this way the meta-analysis was conducted with the help of these five articles. The PRISMA plot of the selection is shown in Figure 2 (40).

### 3.2. Preventive use of pravastatin before the 20th week of pregnancy–Meta-analysis

In our meta-analysis, a total of 5 studies (41–45) were selected (Table 1). Even though most of the articles published birth weights and gestational ages at delivery, since many articles did not publish standard deviations of these data many articles and types of data were left out.

**TABLE 1:** Selected studies.

References	Type of study	Start of treatment (weeks)	Pravastatin (mg/day)	Cases (n)	Conclusions
Costantine (2016) (41)	RCT	12–16	10	20	No identifiable safety risks were associated with pravastatin use in this cohort. Four subjects in the placebo group developed preeclampsia compared with none in the pravastatin group.
Kupferminc (2021) (45)	cohort	12	20	32^a^	Additive treatment with pravastatin to low molecular weight heparin and low dose aspirin may be promising option in cases of previous severe recurrent placenta-mediated complications.
Costantine (2021) (41)	RCT	12–16	20	20	This study confirmed the overall safety and favorable pregnancy outcomes for pravastatin in women at high risk for preeclampsia.
Akbar (2021) (43)	RCT	14–20	40	80	The rate of PE was (nonsignificantly) lower in the pravastatin group. Prophylactic pravastatin was associated with a significantly lower rate of adverse perinatal outcome.
Akbar (2022) (44)	RCT	14–20	20	173	Pravastatin (20 mg bid) significantly reduces the risk of preterm preeclampsia and preterm birth in women at high risk of developing preeclampsia.

RCT, randomized control study.

^a^Retrospective cohort study of 32 women with recurrent severe placenta-mediated complications. Everyone was treated with pravastatin; the previous pregnancy was used as a control group.

We included studies that evaluated the prevention of preeclampsia. Since preeclampsia occurs per definition after the 20th week (46) we only included studies that started the pravastatin treatment before the 20th gestational week.

In our meta-analysis, the evaluated data were the following: the occurrence of preeclampsia, the frequency of NICU admissions, IUGR, and preterm delivery. These data helped to form a clearer picture of the neonatal and maternal benefits of pravastatin use among patients who are high-risk of preeclampsia.

### 3.3. Pravastatin in the prevention of preeclampsia

**FIGURE 3:**
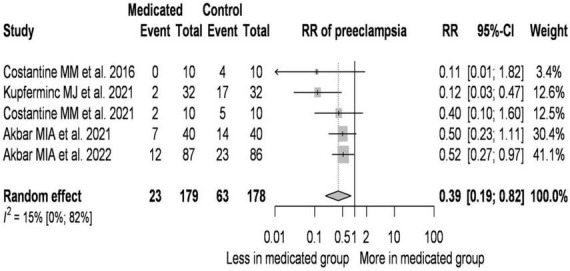
Effect of pravastatin treatment on the prevention of preeclampsia–forest plot.

A total of five studies were selected for analyses covering a total of 357 patients out of which 86 patients had preeclampsia (Figure 3).

On average, the risk ratio (the pooled effect size) of developing preeclampsia was 0.39. The 95% confidence interval of the odds ratio was 0.186 to 0.819, which tells us that the mean effect size in the universe of comparable studies could fall in this range. The between-study heterogeneity expressed as *I*^2^ value was 0.15 (95% CI: 0–0.82), which tells us that 15% of the variance in observed effects reflects variance in true effects rather than sampling error. The variance of true effects (τ^2^) was 0.07 and the standard deviation of true effects (τ) was 0.265. The prediction interval was 0.118 to 1.291. Based on that we would expect in some 95% of all populations comparable to those in the analysis, the true effect size will fall in this range.

The bias due to the low number of cases (41–43, 45) is high. Analysis of the data suggests that a higher pravastatin dose is associated with a higher RR value. If pravastatin treatment was started in a later gestational week compared to the controls, the RR values were higher.

Pravastatin treatment reduced the incidence of preeclampsia by 61% in the pravastatin-treated group compared to the untreated group.

### 3.4. Pravastatin treatment reduces the incidence of IUGR

**FIGURE 4:**
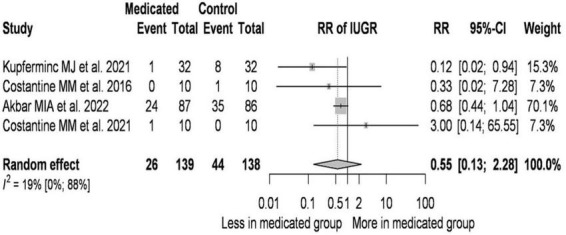
Pravastatin treatment reduces the incidence of IUGR–forest plot.

A total of 4 studies were selected for analyses covering a total of 277 patients out of which 70 patients’ infants were diagnosed with IUGR (Figure 4).

On average, the risk ratio (the pooled effect size) of IUGR was 0.554. The 95% confidence interval of the odds ratio was 0.135 to 2.284, which tells us that the mean effect size in the universe of comparable studies could fall in this range. The between-study heterogeneity expressed as *I*^2^ value was 0.19 (95% CI: 0–0.88), which tells us that 19% of the variance in observed effects reflects variance in true effects rather than sampling error. The variance of true effects (τ^2^) was 0.235 and the standard deviation of true effects (τ) was 0.485. The prediction interval was 0.033 to 9.409. Based on that we would expect in some 95% of all populations comparable to those in the analysis, the true effect size will fall in this range.

Analysis of the data (41–43, 45) suggests that the dose of pravastatin has no role in the incidence of IUGR. In studies where the initial BMI of the treated pregnant women was higher than that of the controls, the RR value was lower.

### 3.5. Effect of pravastatin on preterm birth

**FIGURE 5:**
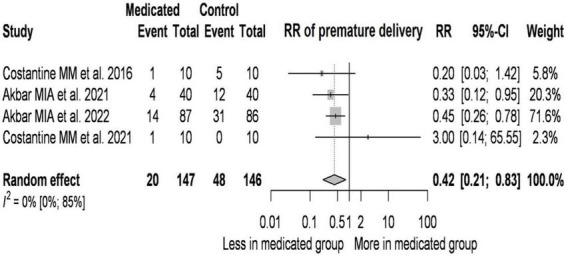
Effect of pravastatin on preterm birth–forest plot.

A total of four studies were selected for analyses covering a total of 293 patients out of which 68 patients gave birth preterm (Figure 5).

On average, the risk ratio (the pooled effect size) of preterm birth was 0.42. The 95% confidence interval of the odds ratio was 0.214 to 0.825, which tells us that the mean effect size in the universe of comparable studies could fall in this range. The between-study heterogeneity expressed as *I*^2^ value was 0 (95% CI: 0–0.85), which tells us that 0% of the variance in observed effects reflects variance in true effects rather than sampling error. The variance of true effects (τ^2^) was 0 and the standard deviation of true effects (τ) was 0.

The prediction interval was 0.149 to 1.18. Based on that we would expect in some 95% of all populations comparable to those in the analysis, the true effect size will fall in this range.

According to our conservative estimate due to the low number of cases (41–43, 45), the dose of pravastatin has no role in reducing premature birth. In those studies where the initial BMI of the treated pregnant women was higher than that of the controls, the RR values were lower. If the pravastatin treatment was started in a later gestational week compared to the controls, the RR values were higher.

### 3.6. Reduction in NICU admission with pravastatin treatment

**FIGURE 6:**
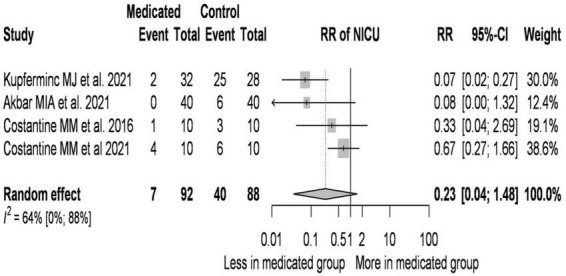
Reduction in NICU admission with pravastatin treatment–forest plot.

A total of four studies were selected for analyses covering a total of 180 patients out of which 47 patients’ infants were admitted to NICU (Figure 6).

On average, the risk ratio (the pooled effect size) of NICU admission was 0.227. The 95% confidence interval of the odds ratio was 0.035 to 1.475, which tells us that the mean effect size in the universe of comparable studies could fall in this range.

The between-study heterogeneity expressed as *I*^2^ value was 0.64 (95% CI: 0–0.88), which tells us that 64% of the variance in observed effects reflects variance in true effects rather than sampling error. The variance of true effects (τ^2^) was 0.679 and the standard deviation of true effects (τ) was 0.824.

The prediction interval was 0.003 to 17.691. Based on that we would expect in some 95% of all populations comparable to those in the analysis, the true effect size will fall in this range.

The most significant change was observed in newborns requiring treatment in the intensive care unit. Newborns of pregnant women receiving pravastatin treatment had 77% reduction in NICU admission compared to untreated pregnant women.

Analysis of the data (41–43, 45) suggests that the RR value decreases as the daily dose of pravastatin increases. If treated patients are older than controls, the RR value is lower. In studies where the initial BMI of the treated pregnant women was higher than that of the controls, the RR value is lower.

### 3.7. Pravastatin in the prevention of preeclampsia after the 20th week of pregnancy

This is the 2nd group studied (preventive use of pravastatin after the 20th week of pregnancy). We found three studies for this group (47–49). Due to the small number of cases, the heterogeneity of the groups included in the study (APS, IUGR, high risk of preeclampsia) and the difference in the start of treatment (24, 20–28, 35,9 weeks, respectively), it was not suitable for meta-analysis. The overall findings of our review have shown positive effects of pravastatin use in preeclamptic women or women with a high risk of preeclampsia. However, the article with the highest sample size (49) did not find significant differences between pravastatin and the control group: Nicolaides et al. at their investigation treated 548 women with 20 mg daily pravastatin and gave a placebo to 543 patients, all the 1,091 patients were high-risk for preeclampsia, and they started their treatment between the 35th and 37th gestational weeks. In the control group 13.62% (74 of 543) and in the placebo group 14.60% (80 of 548) developed preeclampsia, they found no significant between-group differences in gestational hypertension, stillbirth, abruption, neonatal death, or neonatal morbidity either. Although this study indicates that pravastatin has no effect on preeclampsia if it is given after the 35th gestational week, the other studies that started the treatment earlier showed significant, or at least hopeful differences between the placebo and pravastatin groups.

Mendoza et al. in their 2021 article (48) tested 40 mg of daily pravastatin treatment in women whose fetuses developed fetal growth restriction (FGR). 38 women were enrolled in the study, and 19 of them remained as controls. Pravastatin treatment was initiated with the other 19 gravidas between their 20 and 28th gestational weeks and the treatment was carried on until delivery. The authors reported decreased numbers of preeclampsia development in the pravastatin group compared to the control group (6 to 9). NICU admissions were also lower among the neonates whose mothers received pravastatin than in the control group (12 to 15). The mean average birthweight was 1,300 g in the pravastatin group while in the control group this number was 1,040 g.

### 3.8. Pravastatin in the treatment of preeclampsia

This is the 3rd group studied (use of pravastatin for the treatment of preeclampsia). We found three studies for this group (47, 50, 51). Due to the small number of cases, it was not suitable for meta-analysis. The treatment of already-developed preeclampsia with pravastatin has been described in a few studies (50–55).

Ahmed et al. (50) gave 40 mg pravastatin daily to 32 patients and a placebo to 30 patients who developed early-onset preeclampsia and were between the 24th and the 31st gestational weeks: in the pravastatin group sFlt-1 levels lowered, pregnancy was prolonged by 4 days and no babies were lost, meanwhile in the placebo group they registered 3 perinatal deaths. Although the results were promising, the authors did not rule out that the results occurred by chance, but they found no evidence of maternal adverse effects with pravastatin.

Brownfoot et al. in their study published in 2015 (54) also found that pravastatin has beneficial effects in severe preeclampsia: 4 women who were between the 23rd and 30th weeks received 40 mg of daily pravastatin, which resulted in all 4 patients’ disorders stabilized, they reported no fetal or neonatal abnormalities or obvious adverse effects of the mentioned therapy.

We would also like to raise attention to studies where patients received pravastatin in a combination with other medications.

Many pieces of research indicate, that LMWH and low-dose aspirin have a positive effect on the outcomes of preeclampsia (56–60). Now, we would like to present 3 studies where they extended this therapy with pravastatin thus forming a triple therapy of pravastatin, LMWH, and low-dose aspirin.

Lefkou et al. started with a single case report in 2014 (53) where they described a patient who received the triple-therapy of pravastatin, aspirin, and enoxaparin. The mother was free of adverse effects, and she delivered a healthy infant.

After their single case report, the previously mentioned research group published a study (51) where they examined 11 women who had OAPS (obstetric antiphospholipid syndrome), developed preeclampsia and/or IUGR (intrauterine growth restriction), and they generally were in their 21st gestational weeks. All the women who they examined were not responding well to LMWH + low dose aspirin therapy, 7 of them received 20 mg daily pravastatin treatment as an addition, 4 of them–as a control group–remained with the double therapy of LMWH + low dose aspirin. The treated group bore their children generally 98 days after the start of the treatment, the mean birthweight was among the singletons 3,050 g, 1,420 g among twins and 100% of them survived. Meanwhile, the members of the control group gave birth to their children generally 23 days after they refused pravastatin therapy, only one of the four babies survived with a birthweight of 1,200 g. Besides these significant results, they also found that treated mothers had higher NO levels, which can be a logical explanation for better circulations and better outcomes.

The research group also had a study with highly similar designs (47), where 10 patients remained with LMWH + low-dose aspirin therapy, and 11 patients received additional pravastatin therapy. The results showed once again significant differences: the control group’s median delivery time was 26,5 weeks, and 5 of the newborns died (45,5%). In the group, which received triple therapy the median delivery was 36 weeks and all the newborns survived. 8 patients delivered their babies 36 weeks or later (73%) in the pravastatin group, meanwhile, in the control group, all the babies were born preterm.

As it is known, L-arginine is a NO-donor and as such, it has a positive effect on microcirculation (61, 62). A. Jurisic, Z. Jurisic, E. Lefkou et al. studied 15 women who developed uteroplacental dysfunction (54), 5 of them remained untreated but monitored during the pregnancy, the other 10 patients received 40 mg of pravastatin daily, and 1.5 g of L-arginine and the differences between the 2 groups were significant, without a doubt. The untreated patients’ mean delivery time after the diagnosis was 26 days, meanwhile, the treated group’s mean delivery time was 119 days. The treated mothers’ babies’ mean birthweight was 3,050 g, none of them had to be admitted to NICU, and no neonatal complications occurred, meanwhile the mean birthweight among the untreated mothers’ babies were 650 g, all had to be admitted to NICU, 2 of them were lost. 40% of the untreated mothers developed preeclampsia, meanwhile, 10% of the treated mothers developed the disease.

Furthermore, the study which was published in 2021 by Saito et al. (55) presents two cases where preeclamptic women with a history of APS and SLE received pravastatin treatment.

Even though the presentation of two cases is not representative and the results were not compared with control groups, both cases–compared to their previous respective pregnancies where pravastatin therapy was not induced–present evident improvements: Both mothers gave birth to healthy infants while previously one of them had one, while the other had two miscarriages.

Moreover, no maternal or adverse fetal outcomes were described in this study with the usage of pravastatin.

## 4. Discussion

### 4.1. Results of the meta-analysis

In the meta-analysis we evaluated the prevention of preeclampsia before the 20th gestational week with the usage of pravastatin: 5 studies were evaluated and even though its limitations we found promising data about pravastatin reducing the numbers of neonates born with IUGR, neonatal admissions to intensive care units, the incidence of preterm deliveries.

We also found that women who received pravastatin before the 20th gestational week were less likely to develop preeclampsia compared to the control groups.

### 4.2. Results of the systematic review

We reviewed fourteen studies on the effectiveness of pravastatin in the treatment and the secondary prevention of preeclampsia. The studies that were included were yielding the following results:

(1) Pravastatin treatment should be started between the 12th and 30th gestational weeks, a large sample size study (49) strongly indicated that if the treatment is started between the 35th and 37th gestational weeks it does not prevent the development of preeclampsia. However, all the other studies, which started pravastatin therapy earlier (before the 30th gestational week), reported positive aspects in the treatment and/or prevention of preeclampsia.

(2) According to the available evidence 10 mg of pravastatin has already effect on the prevention of preeclampsia, meanwhile according to the reviewed studies 20–40 mg daily has a positive effect on the prevention and the treatment of preeclampsia. We found no evidence of higher toxicity among the patients who were treated with higher doses.

(3) Pravastatin whether it is given in a double-therapy with L-arginine or in a triple-therapy with LMWH and low-dose aspirin have strong benefits in the treatment of preeclampsia compared to their respective control groups. It can help pregnant women to deliver their children closer to the physiological date thus improving the survival of the infants.

(4) In the 14 reviewed articles, among the 797 patients who received pravastatin therapy there were no fetal or neonatal adverse effects reported and only minor maternal adverse effects occurred (e.g., headache).

### 4.3. Discussion of the studies that were used for the meta-analysis, strengths, and limitations

Studies by Costantine et al. were randomized clinical trials where women who were at high risk of preeclampsia received pravastatin between the 12th and 16th gestational weeks. In their 2016 article (41) 10 women received a placebo and the other 10 women received 10 mg of pravastatin daily. In the control group 4 women developed preeclampsia and 0 in the pravastatin group, their results also concluded that the use of it was safe, and only minor adverse effects occurred.

In their later article (42) again 10 women received a placebo and 10 others pravastatin between the 12th and the 16th gestational weeks. This time the pravastatin group received 20 mg and the differences between the two groups were once again significant. Even though they doubled the daily dose of pravastatin the adverse effects remained mild. We also do maintain that this study had an important limitation: the placebo group’s mean BMI was 36.3 while the pravastatin group’s mean BMI was 25.4, this is a vastly significant difference and there is a possibility that this is the reason behind the fact that the pravastatin group faced favorable outcomes since we know that patients with larger BMI are more likely to develop preeclampsia, preeclamptic symptoms (63, 64).

The INOVASIA study evaluated the prevention of preeclampsia with the usage of 20 mg of pravastatin daily. The treatments were initiated between the 14th and 20th gestational weeks. The research group published several articles on their results: in their 2021 article (43) 40 women were enrolled in the control group and 40 in the pravastatin group, and all were at high risk of developing preeclampsia. They reported non-significantly lower rates of preeclampsia occurrence (7 in the pravastatin group and 14 in the control group) and significantly lower rates of preterm delivery (12 in the control group and 4 in the pravastatin group).

In their 2022 article (44) a total of 173 patients were enrolled, all of them were at high risk of developing preeclampsia; 86 of them were members of the control group and 87 of them belonged to the pravastatin group. In this article, they reported significantly lower rates of preterm preeclampsia occurrence (23 in the control group, 12 in the pravastatin group, *p* > 0.05).

Kupferminc et al. (45) retrospective cohort study (45) where 32 women with previous severe placenta-mediated complications were evaluated and their previous pregnancies were used as controls during their study. The women enrolled in the study received pravastatin treatment on the 12th gestational week. During the control pregnancies 17 cases of preeclampsia were reported, all of them with severe features. However, in the pravastatin-treated pregnancies, only 2 women were diagnosed with preeclampsia and the symptoms were mild. The cases of reported IUGRs (from 8 to 1) and NICU admissions (from 25 to 2) also decreased significantly.

### 4.4. Limitations

Due to the small study number, we could estimate the prediction interval of true study effect sizes with high uncertainty which makes the prediction interval nearly meaningless. Therefore, a very essential aspect of the research question cannot be revealed.

Due to the small number of studies, it was not possible to assess publication bias or perform outlier and influential analyses.

Due to the same authors of studies, the generalizability is highly limited.

Since all the studies which used arginine and pravastatin in combinations were concluded by the same research group the generalizability was low. Even though the articles indicate the combination’s positive effect we do not have enough evidence to decide if the arginine-pravastatin therapy has a synergistic effect in the prevention and/or treatment of preeclampsia. Therefore, we could not perform further comparisons on this topic.

The evaluated studies did not provide sufficient information about the patients’ compliance.

The heterogeneity of the studies was assessed by the Higgins and Thompson *I*^2^ statistics, which indicate a possibility for potentially high heterogeneity as the upper limit of the confidence interval of *I*^2^ was about 80–90% in all cases (although the point estimates were about 10–20%, except NICU, where it was about 65%). Unfortunately, the limited number of studies does not allow us to make a more precise estimate of this kind of between-study heterogeneity. Although we used RCTs in the analyses, the difference in the populations can cause different estimates; but as we have a limited number of studies, we could only make some cautious assumptions regarding the effect of these differences.

### 4.5. Screening in the first trimester of preeclampsia

For the use of prophylactic pravastatin therapy before the 20th gestational week, the first-trimester screening of preeclampsia is essential.

The mentioned screenings are usually conducted with the help of Doppler ultrasound in which case MAP (mean arterial pressure) and uterine artery pulsatility index (UtA-PI) are measured (65). Capriglione et al.’s study highlights that in the first-trimester screening of preeclampsia the PI of the uterine artery does not differ significantly in high and normal-risk pregnancies. However, their study found that MAP is significantly higher in high-risk pregnancies, in terms of preeclampsia (66).

Another important field of preeclampsia screening is based on laboratory biochemical markers. These mentioned biochemical markers are the following: pregnancy-associated plasma protein A (PAPP-A), placental growth factor (PlGF), alpha feto-protein (AFP), human chorionic gonadotropin (hCG), unconjugated estriol (uE3), Inhibin A, soluble-endoglinin (sEng), and soluble Flt-1 (sFlt-1) (65, 67).

Zumaeta et al. used maternal risk factors, MAP, UtA-PI in screenings with the combination of PlGF or PAPP-A: in their study they concluded, that if PAPP-A is used rather than PlGF with the combination of maternal factors, MAP, UtA-PI the same detection rate can be achieved but at a higher screen-positive rate (68).

A large critical review of the currently available articles also indicates that the combination of several markers of the above-mentioned ones is the best way to predict the risk of PE (69).

### 4.6. Potential clinical significance and future directions in pravastatin use in preeclampsia

Currently, the only definitive therapy for preeclampsia is delivery (2, 9, 70, 71). Although a lot of pieces of research indicate that aspirin has a positive effect on the disorder, it is single-handedly not sufficient in many cases (72). Since worldwide 10 to 15% of maternal deaths are associated with preeclampsia there is an urgent need of finding more and more efficient therapies, protocols, and drugs (2, 73, 74). Lately, there is a growing interest in pravastatin therapy in pregnancy, however, most of the published studies were performed on animal models and placental tissues. We felt that there is a need to summarize the available clinical data and evidence thus providing more pieces of information on this therapy in humans.

We also do feel that the evidence and positive results (reduction of preeclampsia incidence, fewer cases of IUGR, NICU admissions, and preterm deliveries) should be evaluated in further clinical studies. Another promising result is that we did not find evidence of perinatal adverse effects with pravastatin use during our research. We maintain that pravastatin could have a role in preeclampsia prevention before the 20th gestational week in future clinical practices.

## 5. Conclusion

In our research, we summarized the currently available studies which used pravastatin in the treatment or prevention of preeclampsia. We found that pravastatin has no effect in the prevention of preeclampsia if it is given after the 35th gestational week, it should be given to the patients earlier (up to the 12th gestational week) to make significant benefits; to lower, stabilize or prevent the symptoms of the disease.

We also found that the incidence of IUGR, NICU admissions, and premature delivery are lower among the neonates whose mothers received pravastatin therapy before the 20th gestational week.

In our study, we found no evidence of major side effects with pravastatin given to pregnant patients after the 12th gestational week. We also do hope that our findings could help to obtain new pieces of information about this new therapy and that it could be the foundation of new clinical trials with pravastatin in preeclampsia.

## Data availability statement

The original contributions presented in this study are included in the article/Supplementary material, further inquiries can be directed to the corresponding author.

## Author contributions

ZK, SV, and AS contributed to the conception and design of the study. LN, BM, and ZK collected data on articles that were relevant to the topic. ZH and DV helped to design the database. ZK, BM, DV, ZH, and LN organized the database. SV, BM, DV, and ZK performed the analysis. DV and ZH made statistical analysis. BM, ZK, and SV wrote the first draft of the manuscript. KR and AS text reviewed, corrected, and supplemented. All authors contributed to the manuscript revision, read, and approved the submitted version.
