# Supplementary figures and images for: Pravastatin in preeclampsia: A meta-analysis and systematic review
Source: Front Med (Lausanne). 2023 Jan 13;9:1076372. doi: 10.3389/fmed.2022.1076372 (PMC9880057; doi:10.3389/fmed.2022.1076372)

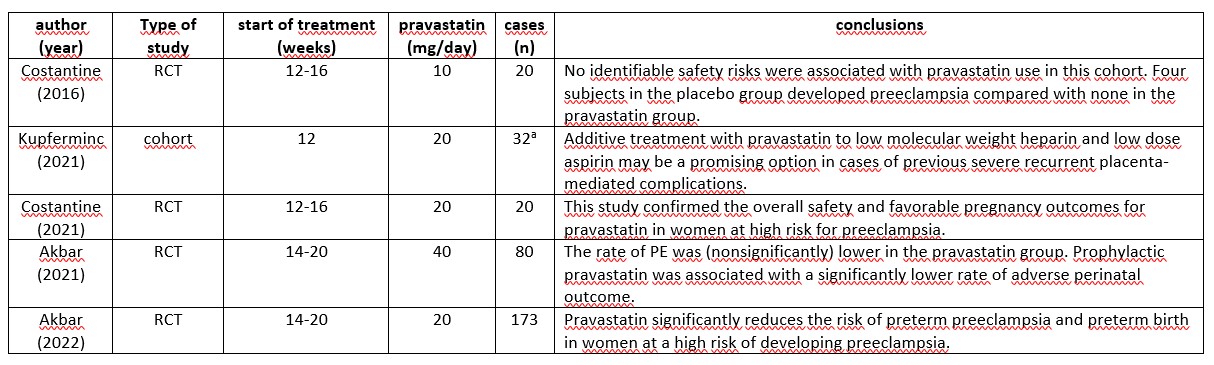

Supplement: Supplementary file 1 [file Image_1.JPEG]
